# An interview with Lyndsey Anderson, 2024 *Epilepsia Open* prize winner for basic science research

**DOI:** 10.1002/epi4.12961

**Published:** 2024-05-13

**Authors:** Aristea S. Galanopoulou

**Affiliations:** ^1^ Neurology & Neuroscience, Albert Einstein College of Medicine Bronx New York USA

## 1 TELL US ABOUT YOURSELF

I grew up in a rural town in Wisconsin and attended the University of Wisconsin for my undergraduate degree in Pharmacology & Toxicology. I received my PhD in Pharmacology from Vanderbilt University investigating pharmacological and genetic treatments for drug‐resistant epilepsies. I then moved to Northwestern University to undertake a postdoctoral fellowship in pharmacogenomics. While there, I continued my work in epilepsy and validated a phenotyping platform in a mouse model of Dravet syndrome. Following my fellowship, I joined the Lambert Initiative for Cannabinoid Therapeutics, a philanthropically‐funded research center at the University of Sydney, to lead the preclinical epilepsy screening program. Using the rigorous pipeline developed at Northwestern, I screened and identified multiple cannabinoids with anticonvulsant potential for the treatment of childhood epilepsies. While I loved my time in Australia, it ended up being bit too far from my family, so I decided to move back to the United States and accepted a position at Praxis Precision Medicines where I am currently an Associate Director of Biology in Discovery Sciences.

## 2 HOW DID YOU BECOME INTERESTED IN CONDUCTING RESEARCH IN THIS FIELD?

I actually sort of stumbled into the epilepsy field. Like many graduate students, I started grad school without knowing exactly what field I wanted to pursue. All I knew was that I was really interested in pharmacology and that I wanted to conduct research that had direct translational impact. Thus, I joined the laboratory of Dr Alfred George, Jr. which was focused on the genetics and pathogenesis of channelopathies. I began a project working on cardiac ion channels but then an opportunity to explore a novel compound in a genetic mouse model of epilepsy arose, so I shifted my attention to neuropharmacology. The more I learned about the brain and epilepsy, the more I enjoyed it. I continue to enjoy exploring the complexities of seizures and strive to translate discoveries to new therapies for epilepsy patients.

## 3 EXPLAIN THE QUESTION YOUR STUDY ADDRESSED, AND HOW YOU DESIGNED IT

In the current study, we investigated whether ictal vocalizations, either as audible mouse squeaks or ultrasonic vocalizations, were characteristics of the *Scn1a* ^*+/−*^ mouse model of Dravet syndrome. We had observed that audible mouse squeaks commonly precede generalized tonic–clonic seizure activity in the Scn1a^+/−^ mice and that during seizure activity the mouse's mouth remains open despite no audible sound being heard. Since communicative behavior of rodents is predominantly in the form of ultrasonic vocalizations, we hypothesized that *Scn1a* ^*+/−*^ mice emitted ultrasonic vocalizations during a seizure. To test our hypothesis, we captured acoustic recordings of *Scn1a* ^*+/−*^ mice undergoing video‐monitoring to quantify spontaneous seizure frequency and then generated audio clips occurring during a generalized tonic–clonic seizure and compared them to nonseizure periods. The audio clips were reviewed to identify and quantify vocalizations as either audible mouse squeaks or ultrasonic.

## 4 WHAT WERE THE RESULTS AND HOW DO YOU INTERPRET YOUR FINDINGS?

We found that spontaneous generalized tonic–clonic seizures in *Scn1a* ^*+/−*^ mice were associated with a significantly higher number of total vocalizations. There was a significantly greater number of audible mouse squeaks with seizure activity. Nearly all the audio clips with seizures contained ultrasonic vocalizations, whereas only about one‐half of the nonseizure clips did. Additionally, the ultrasonic vocalizations emitted in the clips containing seizures were longer in duration and were emitted at a higher frequency. Our results showed that *Scn1a* ^*+/−*^ mice have an “ictal squeak”, analogous to the ictal cry that is common in epilepsy patients with generalized tonic–clonic or focal seizures. Quantitative audio analysis could be further explored as an automated seizure detection tool for the *Scn1a* ^*+/−*^ mouse model of Dravet syndrome and perhaps other mouse models as well.

## 5 WHAT ARE THE NEXT STEPS THAT YOU PLAN TO TAKE, AND WHAT ARE YOUR CAREER GOALS?

Our ultimate vision for this research would be to determine whether ictal vocalizations are a characteristic of other seizure models, especially those where seizure semiology is subtle. Audio‐based seizure detection could then supplement behavioral and video‐EEG monitoring to improve confidence in seizure identification. Further, quantitative audio analysis could be developed as an automated seizure detection tool.

While my career has since progressed from academia to industry, my original motivation to pursue a research path that has direct translational impact remains unchanged. The mission of Praxis Precision Medicines is to translate insights from genetic epilepsies into the development of therapies for patients in need, which aligns well with this personal career goal.

## 6 WHAT DOES THE *EPILEPSIA OPEN* PRIZE MEAN FOR YOU, YOUR LABORATORY, RESEARCH INSTITUTE, AND YOUR FUTURE?

Receiving the prestigious *Epilepsia Open* Prize is an incredible honor for me and my co‐authors. This award highlights the epilepsy research being conducted at the Lambert Initiative for Cannabinoid Therapeutics. I am grateful for my time at the University of Sydney Brain & Mind Centre and for all the support I received from my supervisor Professor Jonathon Arnold.

Read the prize winning article “Ictal vocalizations in the Scn1a^*+/−*^ mouse model of Dravet syndrome”.

## 7 CONFLICT OF INTEREST STATEMENT

ASG is the Editor‐in‐Chief of Epilepsia Open. LA is employed currently by Praxis Precision Medicines but this is not related to the contents of this article. No conflicts of interest exist in regards to the contents of this article.

## Data Availability

Data sharing not applicable to this article as no datasets were generated or analysed during the current study.

